# Supplementary material for: Explicit and implicit timing in older adults: Dissociable associations with age and cognitive decline
Source: PLoS One. 2022 Mar 16;17(3):e0264999. doi: 10.1371/journal.pone.0264999 (PMC8926191; doi:10.1371/journal.pone.0264999)
Supplement: S1 Table — (DOCX) [file pone.0264999.s001.docx]

**S1 Table. Descriptive statistics (mean and standard deviation) of our sample.**

|  | **Male** | | | | | **Female** | | | | |
| --- | --- | --- | --- | --- | --- | --- | --- | --- | --- | --- |
|  |  | **Age** | **Level of education** | **MMSE (raw)** | **MMSE corrected** |  | **Age** | **Level of education** | **MMSE (raw)** | **MMSE corrected** |
|  | **N** | **Mean (SD)** | **Mean**  **(SD)** | **Mean (SD)** | **Mean**  **(SD)** | **N** | **Mean**  **(SD)** | **Mean**  **(SD)** | **Mean (SD)** | **Mean (SD)** |
| **Without cognitive decline** | 21 | 72.19  (6.36) | 9.90  (4.46) | 29.29 (0.77) | 28.01  (1.28) | 21 | 72.86  (6.15) | 10.76  (3.87) | 29.38 (0.86) | 27.82 (1.42) |
| **Mild cognitive impairment** | 11 | 73.09  (5.70) | 9.54  (4.65) | 26.22 (0.93) | 24.93  (0.92) | 16 | 71.50  (5.43) | 9.00  (4.99) | 26.59 (0.90) | 25.39 (1.32) |
| **Mild dementia** | 5 | 76.20  (10.21) | 7.80 (3.27) | 22.70 (1.79) | 22.14  (1.14) | 7 | 79.14  (6.99) | 5.14  (1.46) | 21.71 (2.29) | 22.13 (3.14) |
| **Moderate dementia** | 2 | 86.50  (3.53) | 5.00 (0.00) | 16.00 (2.83) | 16.90  (3.53) | 2 | 74.00  (14.14) | 6.50  (2.12) | 12.00 (2.83) | 12.20 (2.54) |

*Note*: MMSE = Mini Mental Examination State (Folstein et al., 1975). MMSE (raw) indicates participant’s performance and MMSE (corrected) indicates the score corrected for age and education (Folstein et al., 1975; for the Italian version, Magni et al., 1996). According to the cut-offs commonly used in the literature, a score between 30 and 28 would define healthy older adults with a normal cognitive functioning; a score between 27 and 25 would indicate the presence of Mild Cognitive Impairment (MCI); a score between 24 and 19 would indicate a mild dementia, whereas a score between 18 and 10 a moderate dementia.
