# Supplementary material for: Explicit and implicit timing in older adults: Dissociable associations with age and cognitive decline
Source: PLoS One. 2022 Mar 16;17(3):e0264999. doi: 10.1371/journal.pone.0264999 (PMC8926191; doi:10.1371/journal.pone.0264999)
Supplement: S2 Table — (DOCX) [file pone.0264999.s002.docx]

**S2 Table. Summary of the model outputs for the explicit timing task from analyses including participants with a proportion of non-given responses lower than .1, .2, .3, or .4, respectively.**

|  | **Proportion of non-given responses** | | | | | | | |
| --- | --- | --- | --- | --- | --- | --- | --- | --- |
|  | *<.1* | | *<.2* | | *<.3* | | *<.4* | |
| **Fixed Effects** | *Odds ratios* | *p* | *Odds ratios* | *p* | *Odds ratios* | *p* | *Risk Ratios* | *p* |
| (Intercept) | 1.04 | 0.495 | 1.06 | 0.303 | 1.06 | 0.25 | 1.07 | 0.193 |
| Interval duration | 1.98 | **<0.001** | 1.99 | **<0.001** | 1.88 | **<0.001** | 1.86 | **<0.001** |
| MMSE | 1.06 | 0.398 | 1.04 | 0.528 | 1.04 | 0.544 | 1.05 | 0.424 |
| Age | 0.95 | 0.444 | 0.98 | 0.774 | 0.97 | 0.62 | 0.98 | 0.625 |
| Interval duration ⨉ MMSE | 1.23 | **<0.001** | 1.22 | **<0.001** | 1.26 | **<0.001** | 1.27 | **<0.001** |
| Interval duration ⨉ Age | 0.89 | **<0.001** | 0.89 | **<0.001** | 0.87 | **<0.001** | 0.89 | **<0.001** |
| MMSE ⨉ Age | 0.93 | 0.265 | 0.92 | 0.166 | 0.92 | 0.176 | 0.93 | 0.225 |
| Interval duration ⨉ MMSE ⨉ Age | 1.05 | **0.015** | 1.04 | **0.031** | 1.04 | **0.034** | 1.04 | **0.013** |
| N _ID_ |  | 65 |  | 72 |  | 78 |  | 82 |
| Observations |  | 11753 |  | 12949 |  | 13814 |  | 14369 |
| Marginal R^2^ / Conditional R^2^ |  | 0.330 / 0.423 |  | 0.331 / 0.422 |  | 0.313 / 0.400 |  | 0.305 / 0.393 |
