# Supplementary material for: Explicit and implicit timing in older adults: Dissociable associations with age and cognitive decline
Source: PLoS One. 2022 Mar 16;17(3):e0264999. doi: 10.1371/journal.pone.0264999 (PMC8926191; doi:10.1371/journal.pone.0264999)
Supplement: S3 Table — (DOCX) [file pone.0264999.s003.docx]

**S3 Table. Summary of the model outputs for the implicit timing task from analyses including participants with a proportion of non-given responses lower than .1, .2, .3, or .4, respectively.**

|  | **Proportion of non-given responses** | | | | | | | |
| --- | --- | --- | --- | --- | --- | --- | --- | --- |
|  | *<.1* | | *<.2* | | *<.3* | | *<.4* | |
| **Fixed Effects** | *Estimates* | *p* | *Estimates* | *p* | *Estimates* | *p* | *Estimates* | *p* |
| (Intercept) | 6.054 | **<0.001** | 6.065 | **<0.001** | 6.095 | **<0.001** | 6.094 | **<0.001** |
| Interval duration | -0.064 | **<0.001** | -0.066 | **<0.001** | -0.067 | **<0.001** | -0.067 | **<0.001** |
| MMSE | -0.163 | **<0.001** | -0.103 | **0.013** | -0.119 | **0.003** | -0.117 | **0.004** |
| Age | 0.084 | **0.017** | 0.085 | **0.019** | 0.107 | **0.002** | 0.107 | **0.002** |
| Interval duration ⨉ MMSE | -0.015 | **<0.001** | -0.011 | **<0.001** | -0.012 | **<0.001** | -0.011 | **<0.001** |
| Interval duration ⨉ Age | -0.004 | 0.186 | -0.007 | **0.006** | -0.008 | **0.002** | -0.007 | **0.004** |
| MMSE ⨉ Age | 0.005 | 0.899 | 0.04 | 0.299 | 0.042 | 0.281 | 0.044 | 0.256 |
| Interval duration ⨉ MMSE ⨉ Age | 0.001 | 0.743 | 0.004 | 0.152 | 0.003 | 0.252 | 0.004 | 0.173 |
| N _ID_ |  | 65 |  | 72 |  | 78 |  | 82 |
| Observations |  | 11619 |  | 12936 |  | 13932 |  | 14605 |
| Marginal R^2^ / Conditional R^2^ |  | 0.175 / 0.616 |  | 0.123 / 0.630 |  | 0.154 / 0.633 |  | 0.148 / 0.630 |
